# Perspectives for computational modeling of cell replacement for neurological disorders

**DOI:** 10.3389/fncom.2013.00150

**Published:** 2013-11-06

**Authors:** James B. Aimone, Jason P. Weick

**Affiliations:** ^1^Cognitive Modeling Group, Sandia National LaboratoriesAlbuquerque, NM, USA; ^2^Department of Neurosciences, University of New MexicoAlbuquerque, NM, USA

**Keywords:** neurogenesis, functional integration, stroke, embryonic stem cells, induced pluripotent stem cells, dentate gyrus, cerebral cortex

## Abstract

Mathematical modeling of anatomically-constrained neural networks has provided significant insights regarding the response of networks to neurological disorders or injury. A logical extension of these models is to incorporate treatment regimens to investigate network responses to intervention. The addition of nascent neurons from stem cell precursors into damaged or diseased tissue has been used as a successful therapeutic tool in recent decades. Interestingly, models have been developed to examine the incorporation of new neurons into intact adult structures, particularly the dentate granule neurons of the hippocampus. These studies suggest that the unique properties of maturing neurons, can impact circuit behavior in unanticipated ways. In this perspective, we review the current status of models used to examine damaged CNS structures with particular focus on cortical damage due to stroke. Secondly, we suggest that computational modeling of cell replacement therapies can be made feasible by implementing approaches taken by current models of adult neurogenesis. The development of these models is critical for generating hypotheses regarding transplant therapies and improving outcomes by tailoring transplants to desired effects.

## Introduction

In addition to describing normal neural function, new computational modeling paradigms have successfully recapitulated various aspects of neurodegeneration and injury. For instance, models of Parkinson’s Disease (PD) have demonstrated overt motor deficits as well as subtle cognitive symptoms due to loss of striatal dopamine and suggested new hypotheses regarding PD as a disorder of altered synaptic plasticity and not simply of motor function (Wiecki and Frank, 2010). Models of ischemic stroke have successfully recapitulated the reorganization of cortical receptive fields (RFs) after lesion, lending credence to a number of hypothesized mechanisms underlying cortical network dynamics (Duch, 2007). In addition, stroke models employing behavioral metrics have also simulated use-dependent recovery of movement strength that closely mimic clinical observations in stroke patients (Reinkensmeyer et al., 2012).

While a major goal of computational neuroscience is to improve therapeutics for neurological disorders, an as-yet overlooked avenue for treatment modeling has been the incorporation of new cells into an impaired network. Cell replacement therapies have shown significant promise in pre-clinical and clinical trials (Lindvall et al., 2012), where multiple sources of stem cell-derived neurons are effective at ameliorating behavioral deficits of disease models including PD, Huntington’s disease, age-related dementia and stroke (Bjorklund and Lindvall, 2000; Koch et al., 2009). Interestingly, while many mechanisms may cooperate to produce transplant-mediated recovery, evidence suggests that functional integration of transplanted cells with existing circuitries is critical for the long-term benefits of cell replacement.

Notably, modeling therapeutics presents an additional challenge beyond simply reversing the effects of the neurological perturbation. Impaired neural circuits are often moving targets, changing themselves continuously in response to their altered state. Likewise, the effects of proposed therapies have their own temporal and spatial dynamics (Figures 2C, D) . This perspective will focus on the current status of cell replacement for neurological disorders, and will utilize the framework from the adult neurogenesis field to provide a template for understanding how the addition of neurons to adult networks can affect overall network function.

We have developed a set of criteria that we believe will be required for an accurate predictive modeling resource (Table 1). We advocate the use of anatomically accurate models of a neural system (I); while such models are considerably more challenging, the value of an abstract model to examine therapies is likely limited. Related, the model should have the capacity to respond to simulated injury or disease in a biologically realistic way, and should be capable of exhibiting response/recovery processes observed *in vivo* (II). Next, the model should provide a readout that maps to a behavioral metric to examine clinical efficacy of a treatment regimen (III). As for the therapy, it is essential that a mechanistic representation of the therapy itself be incorporated (IV), including temporal and spatial dynamics to the extent possible (V). To illustrate how this approach will apply to an existing paradigm we will discuss the computational modeling implications of using cells derived from human pluripotent stem cells (hPSCs) in the context of stroke, as this area has proven a significant target for both modeling and cell replacement.

**Table 1:** **Criteria for computational models of cell replacement**.

I.	Accurate anatomical and circuit-level representations
II.	Response to injury in an experimentally-validated manner
III.	Incorporation of behavioral metric(s) that can be measured clinically in patients
IV.	Transplants should include all relevant physiological and anatomical features.
V.	Transplants should include temporal dynamics of synaptic connectivity and functional maturation.

## Computational modeling of cortical reorganization and stroke

Stroke lesions in humans typically result from damage to, or occlusion of the middle cerebral artery (MCA), which supplies blood flow to the basal ganglia and nearly the entire dorsolateral surface of the temporal and parietal cortices (Mohr et al., 1998; Ng, 2007). Following mild ischemic injury the brain demonstrates a remarkable ability to compensate for lost or damaged tissue by reorganizing peri-lesional regions of cortex (Nudo, 1997). Recovery is correlated with increases in axonal and dendritic sprouting in peri-infarct regions as well as alterations in synaptic strengths between surviving thalamic and cortical neurons (Stroemer et al., 1995; Brown and Murphy, 2008). Recent *in vivo* imaging studies in mice have revealed that peri-infarct sensory regions can “remap” their RFs after stroke, responding to peripheral stimulations that were previously restricted to now-damaged cortices (Brown et al., 2009; Sigler et al., 2009).

Due to the hierarchical nature of the cerebral cortex, its development and reorganization following injury have been significant targets for modeling studies (Willshaw and von der Malsburg, 1976; Armentrout et al., 1994; Reggia et al., 1996). Many early models focused on the formation of topographic maps of sensory cortex through experience-dependent remodeling. Models that incorporated Hebbian learning paradigms could account for the experimental observations of the “inverse magnification” rule, where small areas of peripheral sensory organs had large cortical representations due to innervation density. Interestingly, even these early abstract models based on self-organizing maps (SOMs) successfully predicted the remapping of somatosensory or visual cortices following lesions (Reggia et al., 1996), as well as changes to RFs based on stimulation and deafferentation (Pearson et al., 1987). However, while SOM models can demonstrate comparable recovery, they are likely too abstract to represent subtle aspects of diseases and therapies such as connectivity dynamics following lesion (Butz et al., 2009).

More anatomically-relevant models have developed hypotheses as to the mechanisms underlying alterations in RFs. For instance, models of sensory cortex that incorporate laterally projecting excitatory and inhibitory units demonstrate immediate expansion of RFs near to the lesion site due to disinhibition; units near the lesion site no longer receive lateral inhibitory connections from ablated cells, unmasking weaker afferent synaptic connections (Sober et al., 1997). Increasingly complex models have incorporated neural spiking dynamics, thalamic relay neurons and neurotransmitter receptors to uncover more subtle changes. A report using this model demonstrated two temporal phases during RF reorganization due to peripheral amputation: a fast (millisecond) phase of “dynamic plasticity” based on simple electrical properties after loss of input, and a slower (hours to days) phase in which NMDA receptor-dependent synaptic plasticity allowed for the reorganization of network dynamics to incorporate surviving cortical cells into remaining circuits (Mazza et al., 2004).

One of the key aspects for modeling stroke recovery *in vivo* and *in silico* is an effective measure of behavioral output (Lytton et al., 1999; Rohrer et al., 2002). Using a simple virtual arm simulation with three pairs of abductors and adductors, Goodall and colleagues successfully demonstrated muscle “weakness” in response to acute lesions of somatosensory cortex (Goodall et al., 1997). Similar models have been used to examine the observation of decreased “smoothness” of movement in stroke patients (Rohrer et al., 2002). A recent report incorporated a measure of wrist flexion force as a function of firing rates of efferent motor neurons along with a stochastic local search algorithm to feedback to the circuit after cortical lesion. In this way the authors successfully modeled motor recovery (increased flexion force) as a function of plasticity within residual, fixed pathways, without alterations in structural dynamics (Reinkensmeyer et al., 2012). Thus, existing models that incorporate multi-layered input and output pathways with lateral connectivity, spiking behavior, and behavioral metrics satisfy our first three criteria for assessing cell-replacement interventions (Table 1) and can likely be extended to examine cell replacement relatively quickly.

## Functional integration of stem cell-derived neurons after transplantation

While physical rehabilitation can assist stroke patients with regaining motor function, neither spontaneous recovery nor current intervention strategies provide complete symptom amelioration (Kalra, 2010), and no therapies exist to recover lost tissue following ischemic insult. In recent years, cell replacement therapies have become an attractive option in pre-clinical studies of ischemic injury, demonstrating transplant-mediated behavioral recovery using multiple cell types and animal models (Bliss et al., 2007). hPSC-derived neurons (hPSNs) can ameliorate limb asymmetries and amphetamine-induced rotational behavior in animals with unilateral MCA lesions, the effects of which can be maintained for months after transplantation. Successful pre-clinical studies have prompted a number of phase I clinical studies which verified safety and feasibility for such therapies in stroke patients (Kondziolka et al., 2000, 2005; Bang et al., 2005). While these studies were not designed to demonstrate efficacy, notable improvements were observed in some patients (Bliss et al., 2010).

While multiple mechanisms may underlie transplant-mediated recovery (Lee et al., 2008; Ohtaki et al., 2008; Horie et al., 2011; Oki et al., 2012; Polentes et al., 2012) incorporation of transplanted cells into host circuitry is thought to be critical for long-term benefits (Bjorklund and Lindvall, 2000; Dunnett et al., 2001). PSNs display all basic physiological capabilities of neurons *in vivo*, including voltage-gated currents, spiking, synaptic activity (Muotri et al., 2005; Johnson et al., 2007; Wu et al., 2007), and integration with existing circuitries. Benninger et al. (2003) showed that stimulation of intact perforant path fibers could elicit post-synaptic responses in PSC-derived neurons grown on rat dentate gyrus (DG) within hippocampal slice cultures. Furthermore, optical stimulation of hPSNs expressing Channelrhodopsin-2 (Boyden et al., 2005; Weick et al., 2010) caused rapid alterations in whole network activity of established mouse networks both *in vitro* and *in vivo* (Weick et al., 2011; Pina-Crespo et al., 2012), confirming a reciprocal interaction between graft and host.

With respect to stroke, transplanted cells improve behavioral outcomes, extend processes into brain parenchyma, and express synaptic proteins (Ishibashi et al., 2004; Daadi et al., 2008; Dihne et al., 2011). Importantly, a temporal correlation exists between the maturation of hPSNs and the recovery of lost contralesional motor function (Gomi et al., 2012; Polentes et al., 2012). Lastly, electrical stimulation of endogenous cortical neurons in peri-lesional regions was shown to trigger immediate post-synaptic responses in transplanted neurons in animals with MCA lesions (Oki et al., 2012). Thus, stem cell-derived neurons are capable of reciprocally integrating with host brain tissue either in normal or diseased animals, and are capable of altering network function via synaptic activity.

## Cell type and network function of cortical-like stem cell-derived neurons

As mentioned, a major consideration for modeling therapeutics is the incorporation of an accurate mechanistic and temporal representation of the therapy itself (Table 1; criteria IV and V). Critical features include the proportion and connectivity of neurons with various spiking phenotypes, as well as the temporal maturation of developing neurons. In the case of stroke this means recapitulating spiking phenotypes of afferent sensory, excitatory projection, and inhibitory interneurons (Xing and Gerstein, 1996; Mazza et al., 2004). At least four major classes of neurons exist according to intrinsic spiking patterns in the cerebral cortex (Gupta et al., 2000; Contreras, 2004) including Regularly spiking (RS), Irregularly spiking (IS), Fast spiking (FS), intrinsically bursting (IB), but also include multiple subcategories such as adapting, non-adapting, delayed, accelerating and stuttering (Ascoli et al., 2008). While most cortical excitatory projection neurons are RS neurons, inhibitory interneurons and excitatory neurons of various subcortical nuclei display a range of spiking phenotypes (Llinas and Jahnsen, 1982), which can have significant consequences to information processing capabilities (Jahnsen and Llinas, 1984; Koch and Segev, 2000; Pissadaki et al., 2010).

**Figure 1:**
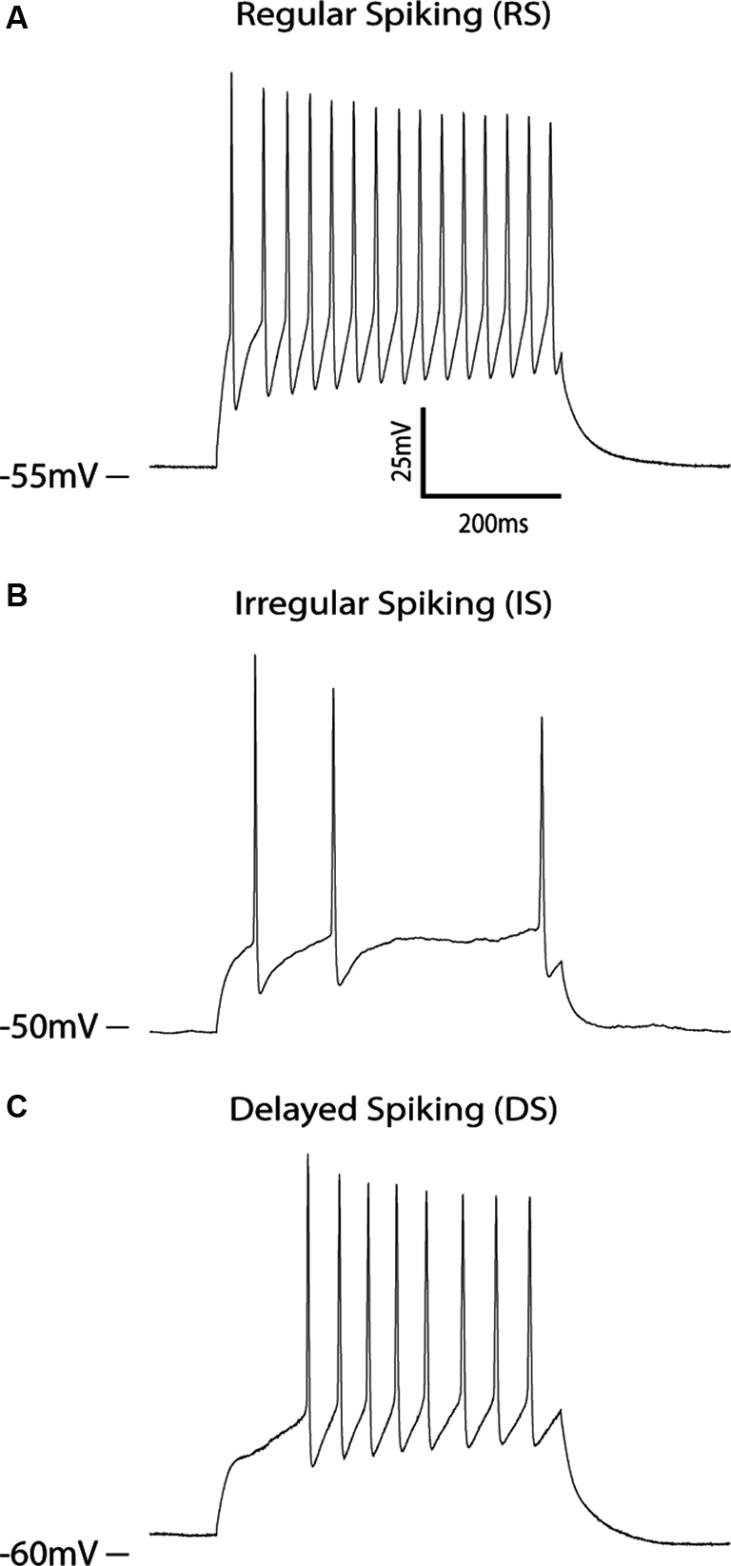
**Spiking properties of hPSNs.** **(A–C)** Voltage clamp traces of three different hPSNs during current injection illustrating various spiking capabilities including RS **(A)**, IS **(B)**, and delayed spiking **(C)**.

Unfortunately, most publications measuring functional properties of hPSNs demonstrate report only RS phenotypes (Wernig et al., 2004; Johnson et al., 2007; Wu et al., 2007). In our hands, hPSNs derived from the WA09 (H9) cell line display primarily RS neurons with a frequency range of 10 to 36 Hz (Figure 1A). In approximately 10% of cells however, we observed delayed spiking phenotype (Figure 1B), while an even smaller minority displayed IS behavior (Figure 1C), with no evidence of IB neurons. However, it is likely that that the dearth of variation is partially due to the immature nature of most hPSNs reported, as many display relatively depolarized resting membrane potentials (RMP) and diminishing action potential (AP) amplitude during current pulses (Johnson et al., 2007).

Interestingly, the development and incorporation of hPSNs into existing neural networks shares many features of adult neurogenesis. Most importantly is the progressive maturation of excitable properties including synaptogenesis (Zhao et al., 2006; Ge et al., 2007), where unitary and network-based synaptic potentials can be observed only after several weeks in the presence of mature cells (Weick et al., 2011; Pina-Crespo et al., 2012), much slower than their rodent counterparts (Johnson et al., 2007). Thus, an additional benefit of computational models may be to examine the incorporation of cells with primate characteristics into a “rodent” network, which will likely affect pre-clinical assessments of cell replacement.

## Lessons from adult stem cells: new neuron incorporation in the dentate gyrus

In order to illustrate the complexity of incorporating new neurons into existing circuits, it is useful to consider systems in which there is ongoing integration of new neurons within the normal brain. Adult neurogenesis in the DG region of the hippocampus provides just such a system (Zhao et al., 2008), with DG neurogenesis producing new excitatory granule cells throughout life. Naturally occurring adult neurogenesis provides the following several important insights into different functional considerations for induced neurogenesis.

**Figure 2:**
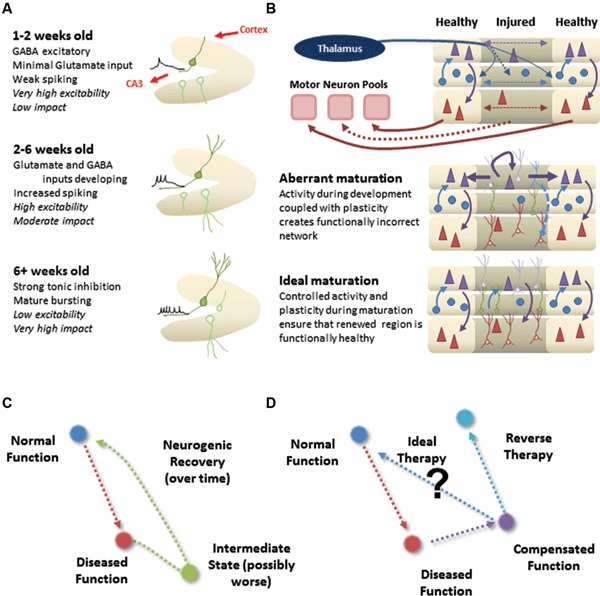
**Incorporation of new neurons into normal or damaged circuitry.** **(A)** Natural context for DG neurogenesis. The DG has a roughly feed-forward architecture, allowing a sophisticated maturation process (different panels). Neurons are born continuously, so there is always a mixed population of neurons at different developmental stages (faded green). **(B)** Therapeutic context for cortical neural replacement. Injured or diseased region may suffer considerable neuronal loss and initiate restructuring of the local network. Proper cell replacement therapy, in which the correct types of new neurons are appropriately positioned in the region, could still suffer from *aberrant maturation* if the unique properties of developing neurons cause affect the functional wiring of the circuit. *Ideal maturation* would instead result in not only the proper neuronal layout, but also an appropriate functional circuit as well. **(C)** Cartoon illustration of how the dynamics of neural maturation can complicate a cell replacement therapy. **(D)** Cartoon illustration of how compensation makes simple reversal of impairments an insufficient strategy for returning system to normal function.

1. *The maturation process is non-trivial*. New neurons pass through several distinct functional stages prior to settling into long-term behaviors (Figure 2A; Aimone et al., 2010). In the DG, new neurons require approximately 2 weeks to start forming functional connections, at which point they begin to form afferent and efferent synapses (Zhao et al., 2006). Over the following weeks, young neurons appear to be considerably more responsive than mature neurons due to their reduced synaptic inputs and different intrinsic properties. They are also highly plastic, both in terms of their dendritic inputs (Ge et al., 2007) and axonal outputs (Gu et al., 2012). From a modeling perspective, it does not appear sufficient to simply represent new neurons as equivalent to existing units. Rather, it will be necessary to understand the temporal dynamics of any cellular replacement process at a fairly exhaustive level to adequately capture the functional implications of the treatment (Aimone et al., 2009; Aimone and Gage, 2011).
2. *Maturation affects other neurons in circuit*. One computationally intriguing observation regarding adult neurogenesis is the direct interaction between new neurons and mature neurons in the network. Electron microscopy studies suggest that young neurons preferentially target existing synapses, both dendritic and axonal, as a prelude to formation of new synapses (Toni et al., 2007, 2008). Thus it appears that synapse formation is not entirely *de novo*, but at least in some cases involves subverting existing synaptic machinery. While synaptic competition presents intriguing computational possibilities for DG neurogenesis, namely a mechanism for young neurons to sharpen tuning curves of mature neurons, this interaction represents a potential concern with respect to adding neurogenesis to other regions.
  A direct interaction between young and old neurons may be suitable for the DG; its primary function is thought to be in facilitating memory encoding, not memory storage. As a result, sharpening RFs over time could be beneficial (O’Reilly and McClelland, 1994; Aimone et al., 2011). In contrast, it is possible that changing otherwise stable RFs in regions involved in motor control or sensory perception would be disruptive. One consideration is that neuronal replacement therapies are generally going to target areas in which neurons have been lost, suggesting that a number of synapses would have been vacated (Lehmann et al., 2005; Butz et al., 2008). Together these possibilities highlight the need to capture the full complexities of the disease and treatment in a theoretical framework (Figure 2B).

## Future perspectives for modeling functional transplantation

The above summary highlights two major computational considerations that must be accounted for when approaching neuronal replacement therapy. First, new neurons do not mature in isolation, and the progression from a progenitor state to a fully functional neuron is complex. While neurogenesis has been evolutionarily preserved in the DG, networks normally lacking neurogenesis may be more sensitive to the addition of neurons. It would not be unreasonable to expect that the addition of neurons could shift the balance of a network out of normal operating bounds, causing either seizures or depressed overall activity (Schneider-Mizell et al., 2010).

Second, the functional displacement of an impairment, either acute or through degeneration, is not static. Rather, compensatory mechanisms are initiated almost immediately. While such compensation can be clinically beneficial it represents an additional challenge for cell replacement. In the time that it takes for a replacement therapy to begin and the neurons to be incorporated, the network itself will have changed; as a result, “reversing” the effects of disease may no longer be sufficient (Figure 2C). Rather, we need a theoretical understanding of the post-disease, steady-state circuit to design an approach to regain a functional equivalence to the pre-disease state (Figure 2D).

What should a model capable of addressing these issues look like? Although not used to model stroke, the three-layer GENESIS model used by Mazza et al. (2004) is among the most neurobiologically realistic as it uses several thousand conductance-based, multi-compartment neurons and accurately reorganizes in response to peripheral lesions. Accordingly, we recommend that the neurogenesis process be appropriately mapped to the primary somatosensory cortex, which could follow the model used in Aimone et al. (2009), whereby the biophysical properties and connectivity of maturing neurons are dynamic over extended time scales similar to hPSNs. This type of hybrid model would provide insight into how remapping could be altered by synaptic connections between hPSNs and host neurons at various levels (e.g., cortical vs. thalamic relay neurons). Alternatively, motor output could be monitored by the creation of a hybrid model using parameters from Reinkensmeyer et al. (2012) and a two-layer GENESIS model to incorporate new neurons into motor cortex, which could allow the system to “learn” to use transplanted cells.

While large-scale, biophysically realistic models are currently time-consuming to construct and computationally expensive to simulate, we envision that future simulation tools and high performance computing capabilities will facilitate such modeling endeavors. We hope that our outlined vision for how to approach these efforts will provide a roadmap for understanding the computational implications of cell replacement therapy. The extent to which insights from adult neurogenesis apply to stem cell therapies remains to be seen, and future models will have to consider not only the proposed therapy but also the unique aspects of the brain region affected, its function, and the disease. Nonetheless, improved modeling tools and approaches in recent years provide computational neuroscience with a unique opportunity to influence the development of an exciting area of therapeutic development.

## Conflict of interest statement

The authors declare that the research was conducted in the absence of any commercial or financial relationships that could be construed as a potential conflict of interest.
